# Pulmonary Sequestration: A Monocentric Case Series Report

**DOI:** 10.3390/jcm13195784

**Published:** 2024-09-28

**Authors:** Michail Galanis, Estelle Sommer, Konstantinos Gioutsos, Thanh-Long Nguyen, Patrick Dorn

**Affiliations:** Department of Thoracic Surgery, Inselspital, University Hospital of Bern, 3010 Bern, Switzerlandkonstantinos.gioutsos@insel.ch (K.G.); thanh-long.nguyen@insel.ch (T.-L.N.); patrick.dorn@insel.ch (P.D.)

**Keywords:** intralobular pulmonary sequestrum, extralobular pulmonary sequestrum, surgical treatment, complications, thoracoscopy, thoracotomy

## Abstract

**Purpose:** Pulmonary sequestration is a rare pulmonary malformation that often necessitates surgical intervention due to potential complications such as recurrent infections or hemoptysis. This case series presents the clinical trajectory of four patients diagnosed with pulmonary sequestration, from initial diagnosis through postoperative care, with a specific focus on the limited arterial supply in two of the cases. **Materials and Methods:** We conducted a retrospective descriptive analysis of four patients diagnosed with pulmonary sequestration who underwent surgical treatment at our institution between January 2013 and November 2022. The affected lung segments were excised via either thoracoscopy or thoracotomy. We evaluated perioperative and postoperative complications, hospital stay duration, histological findings, and the vascular supply of the affected areas. **Results:** Thoracoscopic surgery was initially preferred for all patients, though one required conversion to an open procedure due to technical challenges. Perioperative complications included increased pain and atelectasis. Two patients developed pleural empyema postoperatively, necessitating additional surgical intervention. The overall outcomes were favorable, with appropriate management addressing the complications effectively. **Conclusions:** Pulmonary sequestration, despite its rarity, often requires surgical treatment. Both thoracoscopic and open surgical methods are effective, though thoracoscopic surgery is generally preferred when feasible. The findings underscore the importance of meticulous preoperative planning and vigilant postoperative care to manage and mitigate potential complications.

## 1. Introduction

Bronchopulmonary sequestration (BPS) is a congenital abnormality of the lower airways. This collection of non-functioning lung tissue is separated from the tracheobronchial tree and is supplied by a systemic artery. It can be categorized anatomically into the following subtypes:

· Intralobar sequestration (ILS);
· Extralobar sequestration (ELS);
· Hybrid BPS/congenital pulmonary airway malformation (CPAM) lesions;
· Bronchopulmonary foregut malformation.

This rare congenital anomaly can be detected in about 1 in 10,000 to 35,000 live births. The most common subtype, intralobar sequestration (ILS), is located within a normal lobe and has no visceral pleura. This type often has aberrant connections to bronchi, lungs, or parenchyma, and patients present with recurrent infections. Patients can also be asymptomatic or present with cough, hemoptysis, chest pain, or dyspnea [1]. It usually presents in the first six months of life with respiratory distress or feeding problems. In slightly older children, respiratory symptoms or congestive heart failure may occur. About 10% of cases are asymptomatic.

Extralobar sequestration (ELS) presents early in newborns with respiratory distress, cyanosis, and infection.

## 2. Materials and Methods

From 1 January 2013 to 30 November 2022, six patients with a differential diagnosis of BPS were identified and surgically treated at our institution. In four patients, the diagnosis of BPS was histopathologically confirmed. This case series report presents the results of a retrospective data analysis of these four patients. All patients gave their written consent for further use of their health-related data.

### 2.1. Inclusion Criteria

All patients with histologically confirmed BPS who underwent surgery between 1 January 2013 and 30 November 2022 and signed the general consent form prior to surgery were included.

### 2.2. Exclusion Criteria

- Lack of histological evidence;
- Incomplete data sets;
- Patients who refused to sign the informed consent form.

### 2.3. Description

Case 1:

Background:

A 32-year-old male patient presented with a series of health issues, including persistent cough and frequent lung infections. His medical history was notable for atopic dermatitis and atopic diathesis, characterized by rhinoconjunctivitis allergies and bronchial asthma, which was managed without inhalation therapy. Additionally, he was an active smoker with a daily nicotine consumption of 20 cigarettes.

**Figure 1:**
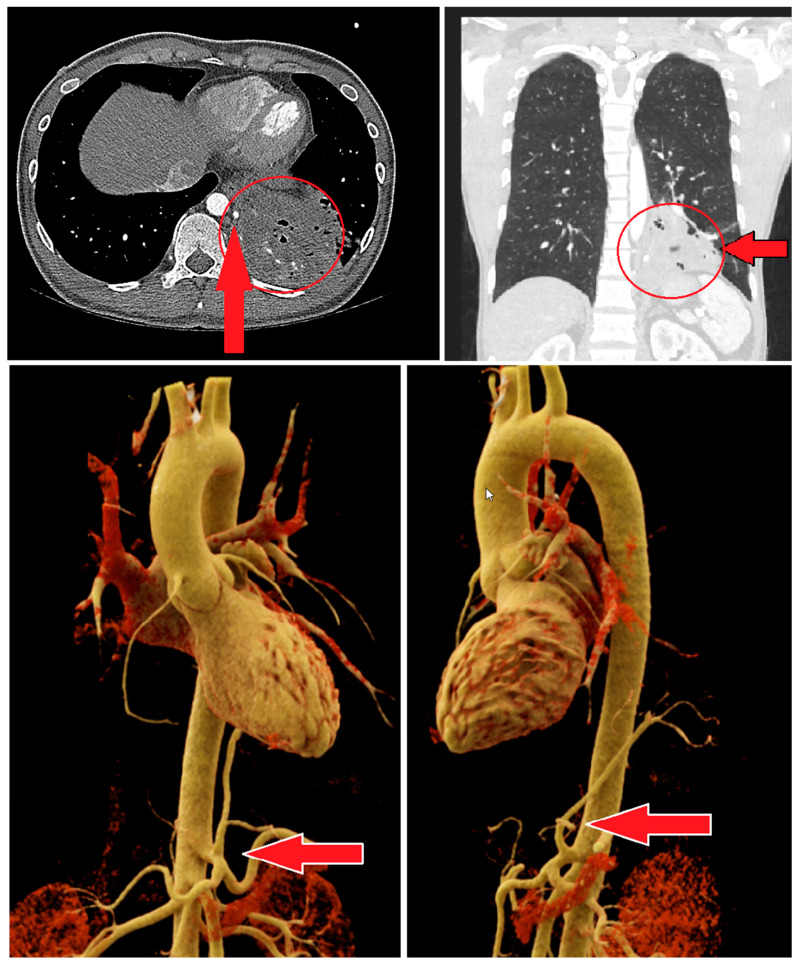
The red arrow indicates the arterial supply to the sequestration of the left lower lobe, which originates from the celiac trunk. The red circle highlights the extent of the sequestration.

Diagnostic Evaluation:

Due to a suspected case of pulmonary tuberculosis, prompted by a radiologically identified cavitary lesion in the left lower lobe, a chest CT scan was conducted. The imaging revealed the presence of a pulmonary sequestration located in the left lower lobe (segments 9–10). This sequestration exhibited multiple cystic components and gas inclusions. The venous drainage of the sequestrum was through peripheral segmental pulmonary artery branches, while the systemic arterial supply originated from a branch of the celiac trunk or the left gastric artery (Figure 1).

Surgical Intervention:

Given the diagnosis of pulmonary sequestration and after excluding specific pathogens, including tuberculosis, surgical intervention was planned. The patient, who was in generally good condition with persistent dry cough and occasional hemoptysis, underwent a thoracoscopic procedure. The surgery involved left-sided adhesiolysis and resection of the pulmonary sequestration. The operation lasted for 219 min and was completed without significant blood loss. Postoperative recovery was initially smooth, and the patient was discharged on the third postoperative day without complications. A follow-up appointment shortly thereafter confirmed that there were no immediate issues.

Complications and Follow-Up:

However, the patient was readmitted on the 24th postoperative day due to a noticeable deterioration in his overall condition. He reported experiencing persistent thoracic pain on both sides, which worsened with breathing, along with new-onset sweating, chills, and loss of appetite persisting for approximately five days. Additionally, the patient experienced significant weight loss, exertional dyspnea, and a mild, intermittent cough. Clinical and radiological evaluations identified a pleural empyema on the left side. On the same day, the patient underwent a thoracoscopic reoperation, which included empyema evacuation and pleural decortication. The microbial analysis of the empyema fluid identified Staphylococcus aureus as the causative pathogen. Post-reoperation, the patient was discharged on the fifth day. His postoperative course was uneventful, as confirmed during a follow-up appointment three weeks later.

Summary:

This case underscores the importance of thorough preoperative evaluation and postoperative monitoring in managing complex pulmonary conditions. Despite the initial success of the thoracoscopic resection, the patient developed a pleural empyema that required additional surgical intervention. The comprehensive management strategy, including careful monitoring and timely reoperation, facilitated a positive recovery outcome.

Case 2:

Background:

A 22-year-old female patient with a diagnosis of scimitar syndrome [2] was evaluated due to recurrent hemoptysis and mild thoracic pain exacerbated by breathing. Scimitar syndrome, a congenital anomaly characterized by the partial or complete absence of the right pulmonary veins, often presents with associated vascular and pulmonary abnormalities.

Diagnostic Evaluation:

A chest CT scan was performed to investigate the patient’s symptoms. The imaging revealed an intralobular pulmonary sequestration localized in the right lower lobe. This sequestration was notable for its unique arterial and venous supply. The arterial blood supply originated from a branch of the abdominal aorta, specifically a vessel branching off at the level of the celiac trunk. Venous drainage was facilitated by high-caliber veins that directly emptied into the left atrium, a significant finding considering the usual drainage routes for pulmonary sequestrations.

Preoperative Management:

Prior to surgical intervention, an interventional embolization was performed to address the intralobular pulmonary sequestration. This procedure aimed to reduce the blood flow to the sequestration, thereby alleviating symptoms such as hemoptysis. Post-embolization, the patient experienced a reduction in hemoptysis but reported new onset of thoracic and abdominal discomfort.

Surgical Intervention:

A thoracoscopic lobectomy of the right lower lobe was then scheduled. The procedure, which lasted 129 min, was performed without significant intraoperative blood loss. The thoracoscopic approach provided the advantage of minimally invasive surgery, reducing potential complications associated with larger incisions.

Outcome and Follow-Up:

The early postoperative period was complicated by severe nausea and vomiting, known as postoperative nausea and vomiting (PONV), which were managed with opioids and antiemetic medications. The patient also developed postoperative pneumonia, which was addressed with conservative treatment and antibiotics. Despite these complications, adequate medical therapy led to a significant improvement in the patient’s general condition.

The patient showed considerable recovery and was discharged home on the 7th postoperative day. Follow-up appointments confirmed an uneventful recovery trajectory, with resolution of the initial symptoms and no additional postoperative issues.

Summary:

This case highlights the complex management of intralobular pulmonary sequestration, particularly in the context of scimitar syndrome. Despite challenges such as severe postoperative nausea and pneumonia, the patient benefited from a well-coordinated approach involving preoperative embolization and minimally invasive surgical techniques. Continued follow-up and appropriate postoperative care were crucial in achieving a positive outcome.

Case 3:

Patient Background:

A 41-year-old female patient was referred by her family physician for evaluation of recurrent hemoptysis, which persisted over a three-day period, accompanied by night sweats and a significant loss of appetite. The patient’s medical history was notable for the earlier diagnosis of a pulmonary sequestration, identified through a PET-CT scan conducted a few years prior while investigating breast carcinoma. At that time, the sequestration was asymptomatic, and no immediate surgical intervention was recommended due to the presence of the primary breast carcinoma and a lack of significant symptoms related to the sequestration.

Diagnostic Evaluation:

The recent onset of hemoptysis prompted a chest CT scan, which revealed two large cavitary lesions in the left lower lobe. These findings were concerning for possible underlying pathology and warranted further investigation. The patient was admitted to the hospital for comprehensive evaluation. A bronchoscopy was performed to assess the airways and rule out active bleeding sources. The procedure did not reveal any active hemorrhage. Bronchoalveolar lavage was conducted to identify potential infectious agents. The results of the lavage showed no evidence of common pathogens such as Aspergillus species or Mycobacterium tuberculosis. However, sputum examination identified a colonization by *Mycobacterium avium*, a rare but significant finding associated with pulmonary disease.

Surgical Intervention:

Given the diagnosis of *Mycobacterium avium* colonization and the presence of large cavitary lesions, surgical resection was indicated to mitigate the risk of further severe hemoptysis and manage the colonization effectively. A uniportal video-assisted thoracoscopic surgery (VATS) was planned for a left lower lobe resection along with radical mediastinal lymph node dissection. The procedure was performed successfully and lasted a total of 139 min. Notably, there was no significant blood loss during the operation. The VATS approach was chosen for its minimally invasive nature, which typically results in reduced postoperative pain and faster recovery.

Outcome and Follow-Up:

The perioperative period was uneventful, with the patient showing a stable recovery. Postoperative pain was managed effectively, and the patient was discharged on the third day following surgery. A follow-up assessment six weeks after the operation indicated an uneventful recovery with no complications. Regular pneumological evaluations, including sputum analysis and bronchoscopy, showed no further evidence of *Mycobacterium avium*.

Summary:

This case underscores the successful management of pulmonary sequestration complicated by *Mycobacterium avium* colonization. The combination of uniportal VATS and targeted postoperative care resulted in a favorable outcome, demonstrating the effectiveness of minimally invasive techniques in addressing complex pulmonary issues. Continued monitoring and regular follow-up were crucial in ensuring the absence of recurrence and maintaining the patient’s overall health.

Case 4:

Background:

A 19-year-old female patient presented with a complex medical history characterized by multiple congenital anomalies and chronic conditions. She had been experiencing recurrent episodes of pneumonia since birth, which were attributed to a prenatally diagnosed Congenital Pulmonary Airway Malformation (CPAM). In addition to CPAM, her medical history included situs inversus totalis, biliary atresia, splenic malformation syndrome, and chronic obstructed nasal breathing. The patient had a history of daily cannabis use, which may have contributed to her overall health status.

Diagnostic evaluation:

At the age of 19, the patient developed an abscessing pneumonia in the right upper lobe. Initial treatment involved a course of antibiotics, which led to partial regression of the lung abscess as observed on X-ray. Despite this initial improvement, the patient continued to exhibit persistent symptoms, prompting a referral to a thoracic surgery consultation for further evaluation and potential surgical intervention.

Surgical Intervention:

Four months after the referral, the patient was admitted to the hospital for surgical treatment. The surgical team initially planned a uniportal video-assisted thoracoscopic resection of the affected right upper lobe. However, during the procedure, dense adhesions from previous infections and the patient’s underlying conditions necessitated a conversion to an open surgical approach. A lateral thoracotomy was performed, allowing for an open resection of the right upper lobe along with a thorough hilar lymph node dissection. During the surgery, the lingula branch, which provided the arterial supply to the affected lobe, was transected using Hem-o-Lok clips. The operation lasted 189 min, with a total blood loss of 400 mL.

Postoperative management included the use of a patient-controlled analgesia (PCA) pump and a ketamine infusion to manage perioperative pain. Despite these measures, the patient experienced severe nausea and vomiting, which required additional antiemetic treatment. The patient’s limited mobility and inability to perform adequate respiratory exercises due to ongoing pain and nausea led to the development of secondary atelectasis. This was addressed with incentive spirometry, chest physiotherapy, and continued antibiotic therapy. Over time, the patient’s pain and respiratory function improved significantly, allowing for the removal of the chest tube. She was discharged home on the fifth postoperative day with a stable condition.

Complications and Follow-Up:

Thirteen days after the open upper lobe resection, the patient returned to the emergency department with worsening general condition and elevated inflammatory markers. Diagnostic imaging, including X-ray and CT scans, revealed a progressive seropneumothorax. The patient was readmitted for further management. A uniportal video-assisted thoracoscopy was performed to evacuate the empyema and to decorticate the right lower lobe. Antibiotic therapy was restarted to manage the infection.

In addition to surgical intervention, the patient received intensive inhalation therapy and respiratory exercises. The use of preemptive pain management strategies contributed to her rapid recovery. She was discharged home on the ninth postoperative day with oral antibiotics.

During a follow-up appointment six days after discharge, the patient reported a favorable course with significant regression of symptoms and improved physical fitness. However, due to persistent symptoms and numbness over the right breast, a diagnosis of post-thoracotomy syndrome was considered. The patient was subsequently referred to a pain clinic for ongoing management and support.

Summary:

This case highlights the challenges in managing a complex patient with multiple congenital anomalies and postoperative complications. Despite an initially successful surgical intervention, the patient faced significant postoperative issues, including a seropneumothorax and post-thoracotomy syndrome. Comprehensive surgical management, postoperative care, and follow-up were crucial in achieving a positive outcome for this patient.

Surgical technique

In two cases, a three-port thoracoscopic procedure was performed, and in two cases, a uniportal/single-port procedure was started. In one case, conversion to lateral muscle-sparing thoracotomy was necessary.

## 3. Results

This case series involves the examination and comparison of four patients with pulmonary sequestration who were treated at our tertiary academic institution. The ages of the patients at the time of their respective operations ranged from 19 to 41 years. Two of the patients sought medical attention due to recurrent hemoptysis, while the other two experienced recurrent infections. In all four cases, the diagnosis of pulmonary sequestration was confirmed through a CT scan.

For the surgical intervention, a thoracoscopic procedure was initially selected for all patients. However, due to extensive dense adhesions, one patient had to undergo a conversion to an open surgical procedure. Despite this, there were no severe intraoperative complications reported in any of the cases.

Following the surgeries, two patients, specifically those aged between 19 and 22, experienced significant exacerbation of pain and severe nausea during the perioperative period. These symptoms were accompanied by the development of pneumonia. It is likely that these issues were exacerbated by limited physiotherapy measures for breathing therapy, which were hindered due to the patients’ pain. This highlights the critical importance of ensuring proper postoperative ventilation of the lungs and providing adequate pain management.

During the early postoperative period, two patients developed pleural empyema, which is a possible complication resulting from chronic infection. Additionally, one patient experienced post-thoracotomy syndrome. These postoperative complications underscore the challenges in managing patients with pulmonary sequestration and the necessity for vigilant postoperative care and monitoring. All data are demonstrated on Table 1.

## 4. Conclusion of the Presentation of Case Reports

Bronchopulmonary sequestration (BPS) is a rare and complex pulmonary malformation characterized by the presence of non-functioning lung tissue that is not connected to the normal bronchial tree but receives its blood supply from an aberrant systemic artery. This abnormality can be detected either in the late prenatal period or in the early postnatal period through ultrasound imaging. Prenatal diagnosis typically involves the use of high-resolution ultrasound to identify abnormal masses or cystic structures in the fetus. If BPS is suspected, further diagnostic imaging is often warranted to confirm the diagnosis and assess the extent of the malformation [3,4,5,6].

Once BPS is detected, follow-up diagnostics usually involve advanced radiographic techniques. Computed tomography (CT) and magnetic resonance angiography (MR-angiography) are commonly used to provide detailed images of the lung and its blood supply. These imaging modalities help in visualizing the exact anatomical location of the sequestration and the associated vascular structures. The literature reports indicate that bilateral BPS, though even rarer, can also occur, presenting additional diagnostic and management challenges.

In most cases, the diagnosis of BPS is established within the first 12 months after birth, often during investigations for other congenital malformations or conditions such as esophageal anomalies [7,8] or congestive heart failure [9]. However, in the absence of severe recurrent respiratory tract infections, BPS may not be identified until later in life, sometimes not until the teenage years or adulthood. In these cases, patients may present with symptoms such as recurrent respiratory infections, cough, and fever, which remain the primary clinical indicators of BPS.

The management of BPS often involves surgical intervention. Surgery is indicated primarily to prevent or address complications that can arise from the malformation, such as recurrent infections or hemoptysis (coughing up blood). The primary surgical approach involves either open thoracotomy or thoracoscopic excision of the affected lung tissue.

The gold standard for diagnosing bronchopulmonary sequestration involves CT or MR-angiography, which are essential for detailed visualization of the affected lung anatomy and vascularization. Intralobar sequestration (ILS) often presents as a solitary nodule or mass, which may be cystic or consolidated [10]. The systemic vascular supply to BPS can vary significantly. In many cases, aberrant arterial branches or branches from the celiac trunk provide the arterial supply, while venous drainage typically occurs via the pulmonary veins [11]. In contrast, extralobar sequestration (ELS) often has venous drainage through larger veins such as the azygos vein, hemiazygos vein, or superior vena cava [1].

Surgical treatment is crucial for managing BPS and preventing complications. Options include open thoracotomy, which involves a larger incision and may be associated with a higher incidence of postoperative pain syndromes, or thoracoscopic surgery, which is minimally invasive and generally associated with less postoperative discomfort. Early surgical intervention is beneficial, as it can prevent the development of respiratory symptoms and chronic inflammatory changes that may result from ongoing infections [12]. Asymptomatic ILS may still warrant treatment to mitigate the risks of developing recurrent pneumonia, lung abscesses, pneumothorax, or even rare malignancies [10,13].

Both thoracoscopic and open surgical approaches are considered safe. However, each has its risks and benefits. Thoracoscopic surgery, while less invasive, may not always be feasible if the sequestration is difficult to access or if extensive adhesions are present. On the other hand, thoracotomy provides better access to the affected lung but comes with increased risks of postoperative complications such as pain and longer recovery times [3].

In summary, BPS is a rare pulmonary malformation that can pose significant diagnostic and therapeutic challenges. Early detection and appropriate management are crucial for optimizing patient outcomes and minimizing complications.

## 5. Discussion

The findings from our small cohort are in agreement with those of two larger case series reviews regarding pulmonary sequestration published by Alsumrain et al. [14] and Marinucci et al. [12], which support surgical resection of the pulmonary sequestration in symptomatic or even asymptomatic patients.

Despite the relatively high rate of postoperative complications in our small cohort, with two out of four cases developing pleural empyema, the long-term results were satisfying. Surgical treatment is indicated in patients diagnosed with pulmonary sequestration due to complications such as recurrent infections or hemoptysis. The gold standard for identifying bronchopulmonary sequestration is CT or MR-angiography, which offer the best opportunities to visualize the anatomy and vascularization of the affected area. ILS can manifest as a solitary nodule or mass, as a (multi-) cystic lesion, or as a consolidation [10].

The systemic vascular supply of BPS varies. Aberrant arterial branches or branches of the celiac trunk supply the BPS with arterial blood, while venous drainage occurs via the pulmonary veins [11]. In ELS, venous drainage mainly occurs via the azygos vein, the hemiazygos vein, or the superior vena cava [1].

Treatment options include open (thoracotomy) or thoracoscopic surgical excision. Early surgical treatment can prevent the development of respiratory symptoms, such as ongoing infections, which can lead to chronic anatomical inflammatory changes [12]. Asymptomatic ILS should be treated to avoid the risk of recurrent pneumonia, abscess, pneumothorax, and various rare types of malignancy [10,13]. Both procedures (thoracoscopy and thoracotomy) are safe, although thoracotomy is associated with a higher percentage of postoperative pain syndromes. In the era of minimal-invasive thoracic surgery and based on the improvement of VATS instrumentation and visualization systems, VATS should be considered the first choice, leaving open approaches for more complex cases when early control of the vessels at the level of the hilum is necessary. Furthermore, the lesion’s dimensions and localization dictate the extension of lung resection in terms of lobectomy vs. sublobar (segmentectomy or wedge-resection). Conversely, recurrent lung or chest infections may promote fibrotic scarring tissue, necessitating a more extensive parenchymal resection.

## 6. Limitations

One of the primary limitations of this case series report is the relatively small number of patients included, which is a direct consequence of the rarity of bronchopulmonary sequestration (BPS) as a clinical entity. Due to the infrequency with which BPS occurs, acquiring a larger sample size within a single institution or study period is inherently challenging. This small sample size limits the generalizability of the findings and may affect the robustness of the conclusions drawn.

Additionally, the retrospective nature of the study introduces several potential biases. Retrospective studies, by design, rely on previously collected data, which may not always be comprehensive or uniformly recorded. As such, there is an increased risk of missing data, inconsistencies in the documentation, and variations in clinical management that were not standardized. These factors can influence the reliability of the results and the ability to make broader inferences about the effectiveness of different treatments or the outcomes of surgical interventions for BPS.

The lack of a control group is another limitation. Without a comparative cohort, it is difficult to determine how the outcomes in this small group of patients with BPS might differ from those in patients with similar conditions who do not have BPS or who are managed differently.

Furthermore, the variability in clinical presentation and treatment responses among the patients highlights the need for a larger, multi-center study to better understand the range of outcomes and to develop more generalized treatment guidelines. The small sample size also limits the ability to conduct subgroup analyses that might reveal differences in outcomes based on specific variables such as age, comorbid conditions, or the presence of concurrent pulmonary infections.

## Figures and Tables

**Table 1:** Demographic and clinical data of all 4 cases.

Case	Age	Gender	Diagnosis	Symptoms	Operation	Operation Time (Minutes)	Length of Hospital Stay (Days)	Bloodloss	Intraoperative Complications	Perioperative Complications	Postoperative Complications	Re-Do Surgery	Length of Hospital Stay (Days)
Case 1	32	M	congenital pulmonary airway malformation; CPAM (Typ II)	cough with yellow/greenish sputum, hemoptysis	thoracoscopic left anatomical segmentectomy IX+X	225	4	not documented	none	drug intolerance	pleural empyema	thoracoscopic pleural decortication	6
Case 2	22	M	intralobar lung sequestrum right lower lobe	haemoptysis, pleuritic pain	thoracoscopic left lower lobectomy	119	7	not documented	none	pain exacerbation, PONV, atelectasis, constipation, incipient SIRS	none	none	
Case 3	41	F	Left lower lobe lung sequestrum	haemoptysis, fatigue	uniportal left lower lobectomy and mediastinal lymph node dissection	139	7	not documented	none	none	none	none	
Case 4	19	F	chronic pneumopathy in congenital pulmonary airway malformation Typ I	recurrent pneumonia	uniportal, thoracoscopic-assisted adhesiolysis, conversion to lateral thoracotomy, open upper lobe resection and hilar lymph node dissection	189	5	400ml	Conversion to thoracotomy for adhesions	pain exacerbation, opioid-induced nausea, atelectasis and incipient pneumonia	pleural empyema, post-thoracotomy syndrome, loss of sensation in the right breast	triportal thoracoscopy with empyema evacuation, decortication of the right lower lobe	9

## Data Availability

The original contributions presented in the study are included in the article, further inquiries can be directed to the corresponding author.

## Abbreviations

BPS	bronchopulmonary sequestration
CPAM	hybrid BPS/congenital pulmonary airway malformation lesions
CT/MR	computer tomography/magnetic resonance
ELS	extralobar sequestration
POVN	postoperative nausea and vomiting
